# Physicochemical Quality and Fatty Acid Profile in the Meat of Goats Fed Forage Cactus as a Substitute for Tifton 85 Hay

**DOI:** 10.3390/ani13060957

**Published:** 2023-03-07

**Authors:** Rafael S. B. Pinheiro, Iasmin M. S. C. Farias, Caroline L. Francisco, Greicy M. B. Moreno

**Affiliations:** 1 School of Engineering, São Paulo State University Julio de Mesquita Filho (Unesp)–Ilha Solteira Câmpus, Ilha Solteira 15385-000, Brazil; 2 School of Veterinary Medicine and Animal Science, São Paulo State University Julio de Mesquita Filho (Unesp)–Botucatu Câmpus, Botucatu 18618-681, Brazil; 3 Câmpus Arapiraca, Federal University of Alagoas, Alagoas 57309-005, Brazil

**Keywords:** cladodes, climate changes, healthy meat, ruminant, sustainability

## Abstract

**Simple Summary:**

The use of cacti, such as forage cactus, in goat feeding is widely practised in Northeast Brazil due to adaptation to climatic conditions, mainly in arid and semi-arid regions. During dry periods, when water is the main limiting factor for the development of most plant species, the growth of forage cactus is not compromised due to crassulacean acid metabolism. However, little is known about the effects of forage cactus on goats’ diet in relation to meat quality. In this study, we evaluated the inclusion of 0, 25 and 55% of forage cactus replacing Tifton 85 hay in the diet of goats and its effects on meat quality. Meat from kids fed 55% of forage cactus showed greater acceptance by consumers. Therefore, it is recommended that Tifton 85 hay be replaced with 55% forage cactus, as it provides lower lipid content and higher monounsaturated fatty acid content in goat meat.

**Abstract:**

Low rainfall in Northeast Brazil is a limiting factor for animal production. Forages that present crassulacean acid metabolism, such as forage cactus, are adapted to the edaphoclimatic conditions of this region, as they lose little water through the stomata. Thus, the objective was to evaluate the physical and chemical quality, fatty acid profile and sensory acceptance of the meat from goats fed forage cactus as a substitute for Tifton 85 hay. Twenty-one uncastrated mixed-breed goats with a mean body weight of 18 ± 0.86 kg and 7 ± 1 months of age were used. A completely randomized design with three treatments and seven replications per treatment was performed. The inclusion of 0 (control), 25 and 55% of forage cactus in substitution of Tifton 85 hay in the diet of the goats was evaluated. The lipid content in the meat of animals fed 25 and 55% of forage cactus was 1.33% and 1.26%, respectively, and was lower (*p* < 0.05) in relation to the meat of animals that received the control diet (1.56%). The inclusion of 55% of forage cactus provided an increase (*p* < 0.05) in the content of monounsaturated fatty acids in the meat (52.71%) in relation to the control meat (37.75%). Sensory analysis differed (*p* < 0.05) between treatments. We recommend replacing Tifton 85 hay with 55% forage cactus, as it presents greater sensory acceptance, and provides lower lipid content and higher content of monounsaturated fatty acids in goat meat.

## 1. Introduction

In Brazil there are approximately 12 million goats and more than 90% of these animals are in the Northeast region [1]. Goats have the ability to survive in extreme environments and are considered the most suitable animal species for the development of arid and semi-arid regions [2]. In the Northeast region, goat farming is a livestock activity of great relevance and social importance [3].

Low rainfall in the semi-arid region of Northeast Brazil is a natural phenomenon that has always occurred and, according to future projections, will be intensified, causing greater aridity and water deficit, compromising the quantity and quality of forage plants, which is one of the factors limiting livestock production in the region [4]. Forage cactus is important for animal production in the Brazilian semi-arid region, due to its physiological adaptability to the edaphoclimatic conditions of this region, photosynthetic efficiency in water use, acceptability by animals, high dry matter digestibility and high-water content, making it an excellent source of energy because it is rich in non-fibrous carbohydrates [5]. Forage cactus is eleven times more efficient in terms of water use than other forage plants used in animal feed [6]. Studies in semi-arid and arid areas in other regions of the world have demonstrated the potential of forage cactus as food and water alternatives for ruminant production [7,8,9,10,11,12,13].

The area of Northeast Brazil is approximately 1,561,177.8 km^2^, equivalent to approximately 18% of the national territory. Most of the region’s territory is under the influence of a semi-arid climate, characterized by severe water deficit (low annual rainfall between 200 mm and 800 mm), irregular rainfall distribution throughout the year, high evaporation rate, low humidity, high surface water runoff and high average annual temperature, above 26 °C [14]. Since 9 million goats are raised in this semi-arid region of Brazil, mainly in extensive systems in natural pastures (predominantly of caatinga vegetation), with the objective of producing meat, milk and skin, this presents significant economic value for the region.

Tifton 85 hay is one of the most commonly used roughages in the Brazilian semi-arid region by rural producers, especially when there is a shortage of forage for ruminants. The forage cactus, despite also being widely used in the dry season, has as a limitation the need for manual and daily cutting before feeding the animals, which implies a greater demand for labor. Few studies have evaluated whether forage cactus, in different proportions in the diet of goats, alters the fatty acid profile of their meat. Most studies on this subject were carried out with sheep [15,16,17]. Furthermore, it is important to highlight that forage cactus is widely used in animal feed in different parts of the world and the Northeast region of Brazil has the largest area of cactus planted on the planet [18].

The consumption of goat meat is increasing, and quality is a decisive parameter as it determines the interest and acceptability of the product by consumers [19]. The appearance, tenderness, juiciness and flavor are factors that lead the consumer to appreciate or not appreciate the meat [20]. Goat meat has a low percentage of cholesterol, low content of saturated fatty acids and high percentages of polyunsaturated fatty acids, which is considered a healthier alternative compared to other types of animal protein [21]. In addition, the distribution of fat in goat meat is different when looking for healthier food, as goats tend to deposit most of their fat in the abdominal cavity [22].

However, there are few studies that evaluate the effects of forage cactus on goats’ diet in relation to meat quality. Therefore, our hypothesis is that forage cactus replacing Tifton 85 hay provides improvements in goat meat quality characteristics due to the high content of non-fibrous carbohydrates and total digestible nutrients, in addition to positively altering fatty acids, providing better quality meat. Thus, the objective was to evaluate the physical and chemical quality, fatty acid profile, and sensory acceptance by consumers of the meat of goats fed forage cactus as a substitute for Tifton 85 hay.

## 2. Materials and Methods

### 2.1. Approval from the Animal Ethics Committee

The study was approved by the Ethics Committee on the Use of Animals of the Federal University of Alagoas, protocol No. 66/2018 and by the Human Ethics Commission under number 040640/2020.

### 2.2. Collection of Meat Samples

The meat samples came from the field experiment performed with goats and conducted at the Federal University of Alagoas—Campus Arapiraca, located in the municipality of Arapiraca, belonging to the State of Alagoas, Brazil, with latitude 9°45′09″ and longitude 36°39′40″ at 280 m above sea level. The climate of the region is tropical savannah (As), characterized by a drier period in the summer, according to the Köppen classification [23].

The *Longissimus lumborum* muscle was used from 21 goats, non-castrated, mixed breed, and slaughtered with a mean body weight of 18 ± 0.86 kg and 7 ± 1 months of age, after a period of finishing in feedlot of 90 days for all animals in this study.

The roughage:concentrate ratio was 80:20 based on dry matter. The chemical composition of diets ingredients in a dry matter basis it is shown in Table 1. The diets consisted of two levels of replacement of Tifton 85 hay (*Cynodon* spp.) by forage cactus (*Nopalea Cochenillifera* Salm-Dyck): 25 and 55% and a control diet without forage cactus in the composition (Table 2). The diets were formulated according to [24], being isoproteic (14.0% crude protein) and isoenergetic (2.3 Mcal/kg DM of metabolizable energy). The roughages were ground daily in a forage crusher (Trapp, model JK 700, Jaraguá do Sul, Brazil), and the particle sizes used were 1 cm and 3 cm for Tifton 85 hay and forage cactus, respectively. The roughages were mixed with the concentrate and provided *ad libitum* to the animals at 08:00 and 16:00, with 20% of the surplus being allowed. The average dry matter intake of the animals in the control diet was 377.49 g/day and the diets with forage cactus inclusion levels of 25 and 55% were 498.46 and 443.10 g/day, respectively. There was no difference for the dry matter intake of the diets between the animals.

After the finishing period, the kids were transported to the slaughterhouse 83 km away, where 78.3 km were paved road in good condition and 4.7 km were dirt road. At the slaughterhouse, the animals underwent a solid fasting period of 16 h, and the animals were stunned by brain concussion using the non-penetrating method [25]. All animals were slaughtered on the same day. After slaughter, the carcasses were cooled for 24 h at 4 °C in a cold chamber. Then the carcasses were sectioned (right and left sides) and the *Longissimus lumborum* muscle was removed from the right and left sides of the carcass of each animal, identified, individually vacuum-packed (Selovac, model 200B, São Paulo, Brazil) in polypropylene packages and stored in a freezer at −18 ± 4 °C (Metalfrio, model DA550, São Paulo, Brazil).

The samples of ingredients and diets were collected weekly throughout the experimental period. The analysis of the chemical composition and fatty acid profile of the diet ingredients based on dry matter (Table 1) was performed from the sum of six samples representing the beginning, middle and end of the experiment (two samples per period), following the recommendations [26], with the dry matter content expressed in g/kg of natural matter for the other items of the diet. Chemical composition of the diet ingredients was expressed in g/kg DM (dry matter). The fatty acid content of the diet was performed according to the described methodology [27].

The samples were packed in a thermal box with dry ice and transported to São Paulo via air transport lasting 3 h to the Laboratory of the State University of São Paulo. Then, the samples were stored in a horizontal freezer (Metalfrio, model DA550B2352, Brazil) at −25 ± 2.3 °C until the analysis was performed.

### 2.3. Physical Quality of Meat

The dimensions of the *Longissimus lumborum* muscle on the right and left sides of the carcass were on average 14 cm long, 6 cm wide, and 1.5 cm thick. The muscles were sectioned while still frozen with a band saw (Lynus, model 17652, Araquari, Brazil) in the cranial (9 cm long) and caudal (5 cm long) portions. Subsequently, the samples were thawed for 24 h in a BOD (Marconi, model M4415, Piracicaba, Brazil) at a temperature of 4 °C. The *Longissimus lumborum* from the right side of the carcass was divided into two portions. The cranial portion was used to evaluate pH, color, water retention capacity, cooking weight loss, shear force and fatty acid composition; the caudal portion was used to evaluate the chemical quality of the meat. For the sensory analysis of the meat, the *Longissimus lumborum* from the left side of carcass was used.

### 2.4. Color

The 9 cm long cranial portion was unpacked and cut into 3 cm long subsamples so that the color could be analyzed after exposure to air for 30 min. Instrumental color was evaluated using a portable colorimeter (Konica Minolta, model CR-400, Osaka, Japan), with an opening of 8 mm of open cone and D65 illuminant. The equipment was previously calibrated with a white ceramic standard and a reading was performed in each subsample, totaling three measurements per sample. The averages of the color parameters were expressed according to the CIE standard, being L* luminosity, a* red intensity and b* yellow intensity (Commission Internationale de 1′ Eclairage—CIE, 1976). The tilt angle (h*) and chroma (C*) levels were calculated as: H° = tan −1 (b*/a*) × (180/π) expressed in degrees and C* = √(a ^(2+)) b^2, respectively, according to the recommendations of the American Meat Science Association [28].

### 2.5. pH

The pH was analyzed with an apparatus to measure pH (model HI99163, Hanna instruments, Póvoa de Varzim, Portugal), coupled with a glass electrode and a smooth penetration tip. The peagometer was previously calibrated using two buffer solutions of pH 4.0 and 7.0 at room temperature (23 °C ± 2). Before and after each reading, the electrode was cleaned with distilled water and a cotton towel.

### 2.6. Water Holding Capacity

To determine the water retention capacity, approximately 0.5 g of the cranial portion of the *Longissimus lumborum* muscle was used. The samples were placed between two circular filter papers and these between two smooth surfaces of polypropylene material and a 10 kg weight was placed on the surface for 5 min. Next, the samples were weighed and the amount of water lost was calculated as the difference in weights [29]. The results were expressed as the percentage of water retained in relation to the initial weight of the samples. All samples were performed in triplicate.

### 2.7. Cooking Losses

The samples were individually weighed, packed in polypropylene, identified, and sealed for cooking in a water bath (Solidsteel, model SSD 5L, Piracicaba, Brazil). The equipment was previously heated until reaching a temperature of 85 °C, at which time the samples were immersed for cooking for 20 min at 75 °C. Then, the samples were removed from the water bath, unpackaged, cooled to 25 °C, and weighed again. The difference in the weight of samples before and after cooking was expressed as a percentage [30].

### 2.8. Shear Force

The samples used for cooking losses were the same as those used to determine the shear force. The meats were cut into cubes of approximately 1 × 1 cm parallel to the meat fibers [31], and the cubes were cut in the transverse direction of the muscle fibers, using a texturometer (CT3 25K, model Brookfield 15W, Middleboro, MA, USA), equipped with a 1 mm thick Warner–Bratzler blade, with a capacity of 25 kg, 200 mm disconnector speed per minute, and 25.0 mm platform distance. Results were expressed in Newtons (N).

### 2.9. Chemical Composition

The *Longissimus lumborum* samples were ground in a processor (mallory, model B91201652, Itapevi, Brazil) and evaluated in duplicate. Moisture, protein, and mineral content were performed according to the Association of Official Analytical Chemists [32]. Lipid extraction was performed using the cold technique with 20 g of meat and a methanol: chloroform: water solution [33].

### 2.10. Fatty Acid Profile

After lipid extraction using the cold technique, the samples were submitted to trans-esterification/esterification of triglycerides to obtain methyl esters through area normalization [27]. The esters were analyzed by gas chromatography with a split/splitless inlet. A microliter of sample was injected into the inlet (Shimadzu, model GC-2010, Plus GC-gas chromatograph, California, USA) with a flame ionization detector at 250 °C, and a fused silica capillary column (Agilent J&W DB-FATWAX Ultra inert, 30 m × 0.25 mm, 0.25 µm, Agilent 7890B, Santa Clara, USA). The carrier gas was hydrogen with a flow rate of 1.2 mL/min. Fatty acids were identified by comparing the retention time of the methyl esters of the fatty acid standards of the samples (Sigma, FAME Mix, C4–C24) and calculating the peak areas determined by the GC Solution software, version 2.4.1.91 (São Paulo, Brazil). The results were expressed as a percentage of area of each acid over the total area of fatty acids (%).

### 2.11. Sensory Analysis

All samples were cut to a size of 1 cm^3^ before cooking the meat. The meats were cooked on an electric grill (George Foreman^®^ 1100-W), preheated for 10 min. The temperature of the samples was monitored by a digital thermometer (Incoterm, model 766202000, Porto Alegre, Brazil) and when the internal temperature of the meat reached 75 °C in approximately 3 min, the sample was turned on its side in the rack. At each change of experimental treatment, the grill was sanitized and the same cooking procedure as the meat was performed.

The sensory analysis involved 91 consumers who appreciate goat meat [34]. The sensory acceptance test was performed using a nine-point hedonic scale, considering the following attributes: general appearance, aroma, flavor, texture and juiciness. The nine points on the scale range from 1 to 9, being: 1- I dislike it very much, 2- I dislike it a lot, 3- I dislike it regularly, 4- I dislike it slightly, 5- I don’t care, 6- I like it slightly, 7- I like it regularly, 8- I like it a lot, 9- I liked it very much [35].

### 2.12. Statistical Analysis

Statistical analysis was performed considering a completely randomized design, using diets (three diets) as a fixed effect and seven replications per treatment (seven animals per diet/treatment, totaling 21 animals used in this study). Each replicate within treatment was considered as an experimental unit. Initially, the data were verified for the distribution of normality using the Shapiro–Wilk (W) test, with the UNIVARIATE procedure (version 9.4, SAS Inst. Inc., Cary, NC, USA) and plot command to allow visualization of the distribution of data points (W = 0.90). Suspicious data were identified by the generalized extreme student deviation (ESD) test and then removed. Four data were identified as suspect and excluded from the control dataset (80% Tifton-85 hay + 20% concentrate), for the variables: pH (*n* = 1), mineral matter (*n* = 1), and, lipid (*n* = 2). One datum was identified as suspect and excluded from the 55% treatment dataset (55% palm + 25% Tifton-85 hay + 20% concentrate), for the moisture variable (*n* =1).

Data on meat variables (color, pH, water holding capacity, cooking losses, shear force, chemical composition) were analyzed using the GLIMMIX procedure (version 9.4, SAS Inst. Inc., Cary, NC, USA), with the Kenwardroger command to adjust the denominator degrees of freedom (DDFs) for reduced samples [36]. The model included diets as a fixed effect, with the term animal (diets) used as a random variable.

Meat fatty acid content data were analyzed by a generalized linear mixed model as logarithmic of the data by the GLIMMIX procedure (version 9.4, SAS Inst. Inc., Cary, NC, USA). The model included diets as a fixed effect, with the term animal (diets) used as a random variable. The results were transformed and presented in real values of fatty acid contents within the different diets.

All results obtained are reported as means of least squares and separated by the least significant difference (Least Significant Difference–LSD) according to diet. For all analyses, significance was established at *p* ≤ 0.05, a tendency was considered with 0.05 < *p* value < 0.10.

The normality of sensory data totaled 91 repetitions per variable analyzed and was verified by the Shapiro–Wilk test (W ≥ 0.90). Subsequently, the data were analyzed by a mixed generalized linear model, using the GLMMIX procedure (version 9.4, SAS Inst. Inc., Cary, NC, USA). The model included treatment as a fixed effect and the term repetition (treatment) as a random effect. The means were presented according to the treatment and compared by the mean of least squares (LSMeans), with significance considered if *p* ≤ 0.05. Statistical analyses were performed in SAS MIXED with the aid of the statistical package [37].

## 3. Results

### 3.1. Physical Quality of Meat

The physical quality parameters of the meat were not (*p* > 0.5) influenced by the inclusion of forage cactus in the diet (Table 3), except for the shear force, which had a tendency (*p* < 0.08) for greater tenderness in the meat of the animals fed 25 and 55% forage cactus compared to the meat of the animals of the control treatment. There was no difference (*p* > 0.05) for the pH values of the meat, with an average value of 5.93.

Cooking weight loss and meat water retention capacity were not influenced (*p* > 0.05) by diets, with mean values of 30.44% and 78.58%, respectively (Table 3). The meat color parameters were not influenced by the diet (*p* > 0.05) with mean values of luminosity, red and yellow intensity of 41.68, 19.86 and 7.85, respectively. The hue angle and the meat saturation index did not differ (*p* > 0.05) in relation to the diets fed to the goats, with mean values of 21.43 and 21.43, respectively (Table 3).

### 3.2. Chemical Composition of Meat

The moisture content of the meat of the kids that received 55% of forage cactus was lower (*p* < 0.05) compared to the meat of the animals that received the control diet (Table 4). Animals fed inclusion of 25% forage cactus in the diet showed no significant difference in relation to the meat unit value when compared to the other experimental treatments. The values of proteins and mineral matter in the meat presented mean values of 20.48% and 1.15%, respectively, and were not influenced (*p* > 0.05) by the studied diets (Table 4).

Meat from animals fed a diet with 25 or 55% forage cactus had lower (*p* < 0.05) lipids content compared to meat from animals fed the control diet. The animals that received 55% of forage cactus in the diet showed lower values (*p* < 0.05) of lipids in the meat compared to those fed 25% of forage cactus (Table 4).

### 3.3. Fatty Acid Profile

The content of total saturated fatty acids in goat meat showed no difference (*p* > 0.05) between the diets (Table 2), with an average value of 40.08% (Table 5).

The content of total monounsaturated fatty acids was higher for meat from goats fed 55% of cactus in relation to control with values of 52.17% and 37.75%, respectively (Table 5). The total polyunsaturated fatty acids were influenced (*p* < 0.05) by the inclusion of forage cactus in the goats’ diet, with values in the meat of 21.44% for animals in the control diet and 6.90% for the diet of the animals fed 55% forage cactus. The polyunsaturated fatty acids with the highest concentration (*p* < 0.05) in the meat of the animals fed the control diet were C18:2cisn6, C20:4n6 and C20:5n3 compared to those fed 55% forage cactus (Table 5).

The omega-6:omega-3 ratio in the meat (Table 5) showed a tendency (*p* < 0.08), being higher in the meat of the animals fed the control diet compared to those fed 55% forage cactus. The monounsaturated: saturated ratio showed a higher (*p* < 0.006) value for the meat of animals fed 55% cactus forage in relation to the control. The polyunsaturated: saturated ratio was higher (*p* < 0.001) for the meat of the animals on the control diet compared to those fed 55% forage cactus (Table 5).

### 3.4. Sensory Analysis of Meat

The general appearance, aroma, flavor and juiciness of meat from animals fed 25 and 55% forage cactus showed higher scores in the sensory analysis, indicating greater acceptance by consumers (*p* < 0.05) compared to meat from animals who consumed the control diet (Table 6).

There was no difference (*p* > 0.05) in relation to sensory attributes between the levels of 25 and 55% of forage cactus, except for the texture. However, this variable did not differ (*p* > 0.05) between the meat of the animals in the control diet and the meat of the animals that received 25% of cactus in the diet (Table 6).

## 4. Discussion

### 4.1. Physical Quality of Meat

The use of cacti, such as forage cactus, in goat feeding is widely practised in Northeast Brazil due to adaptation to climatic conditions, mainly in arid and semi-arid regions. However, there are few studies in the literature that evaluate the inclusion of forage cactus in the goat diet and the effects on meat quality parameters. Therefore, research with the goat species is needed to assess the effects of diet on meat quality.

Animals that consume forage cactus have softer meat than animals fed Tifton 85 hay (Table 3). The higher intake of non-fibrous carbohydrates is related to glycogen synthesis and carcass pH value [38]. However, all pH values from this study (Table 3) are within the normal range (5.5 to 6.0 pH range) for goat meat [39]. High pH values in goat meat are common and reported in the literature [40,41,42], possibly due to the very active behavior of goats in relation to other ruminants, which predisposes them to produce meat with a higher pH [43,44].

Meat unit value is inversely related to fat content, so moisture and fat can also influence meat tenderness [45,46]. The moisture content (Table 4) was not enough to change the values of water-holding capacity and weight loss due to meat cooking (Table 3). The results obtained for the cooking weight losses are in agreement with the value cited in the literature (approximately 35%) for goat meat [47]. In addition, they agree with Oliveira et al. [48], who studied the inclusion of forage cactus in the diet of goats and obtained an average of 28.33% weight loss by cooking in the meat of animals fed 1000 g kg^−1^ of DM of cactus/day.

The values of shear force were lower than those found in the *Longissimus Dorsi* (129.94 N) of crossbred Boer x SRD goats [49], and lower than the *Semimembranosus* muscle (96 N) of goats fed cactus [41], and similar to the result found in the meat of confined goats (72.76 N) in a semi-arid region of India [50]. Meat with a shear force above 98 N is classified as not very tender and of low consumer acceptance; between 78 and 98 N is moderately tender, and considered acceptable; and below 68 N as tender with high sensory acceptance [40,51,52]. The shear force of meat from animals that received the control diet (Table 1) was within the range of moderate tenderness (78 to 98 N), while the average shear force for meat from goats that received 25% or 55% of cactus inclusion in the diet were classified as very tender meat, with a value lower than 68 N.

Probably because there were no differences in the physical quality variables of the meat (Table 3), there was no change in color. All experimental diets contained pigmenting ingredients, including both the control diet, with 80% Tifton 85 hay rich in carotenoids such as lutein [53], as well as forage cactus, with approximately 29 μg of carotenoids/100 g of cladodes [16,54,55]. In this case, the inclusion of 25 or 55% of forage cactus was not enough to influence the intensity of yellow in the meats (Table 3). The luminosity content is positively correlated with the water-holding capacity. However, the water-holding capacity content was similar (*p* > 0.05) and, therefore, did not change the luminosity value of the goat meat, with an average of 41.68 [16].

For the hue angle, the closer to 0° the redder the color, and the closer to 90° the more yellow the flesh color. The saturation index indicates the intensity of the color [26]. High values of shade angle added to low values of saturation index, resulting in meat with brown coloration due to myoglobin oxidation [56].

### 4.2. Chemical Composition of Meat

The goat meat has a low lipid content with a distinct distribution in relation to other ruminant species, since the main lipid deposit in goats is in the abdominal cavity and not subcutaneous and intramuscular, as occurs in cattle and sheep [57,58]. These results (Table 4) of the low content of lipids in the meat of kids fed on cactus agree with Mahouachi et al. [59] who observed a decrease in the amount of lipids from 142 to 101 g/kg in the meat of kids fed on cactus. Forage cactus influenced the lipid content (Table 4) in goat meat, and this influence may favor consumers who prioritize the consumption of protein with low lipid content or who seek healthier foods.

The averages for the moisture values are within the recommended range for raw meat, which varies from 70 to 77% [42]. The values of protein and mineral matter in sheep meat were also not influenced by forage cactus at the levels of 25, 50, 75 and 100% inclusion [60].

### 4.3. Fatty Acid Profile

The inclusion of forage cactus (0, 150, 300, 450 g/kg) in the diet of lambs promoted an increasing linear effect on monounsaturated fatty acids (MUFAs) [17], confirming the results of this study (Table 5) for meat from animals fed 55% forage cactus. Monounsaturated fatty acids have been associated with a reduction in the risk of cardiovascular diseases, as they decrease the concentrations of low-density plasma lipoproteins, responsible for the increase in cholesterol [15]. The PUFAs also have beneficial effects on blood pressure [61].

In the present study, the C18:1cisn9 fatty acid had the highest content among the monounsaturated fatty acids, being considered a thermodynamically stable fatty acid resistant to the oxidative processes of food, which increases the shelf life of meat products [62]. In the literature, an increase in the C18:1 content was observed in the meat of sheep and goats fed 300 g DM/day of forage cactus instead of barley, with an average of 32% in sheep meat and 43% in goat meat, corroborating data from other studies [63] and from the present work (Table 5). The forage cactus diets contained a higher percentage of C18:1 acid (Table 2) which provided a higher concentration of this acid in the meat of animals fed 55% forage cactus in the diet compared to the control diet (Table 5).

In the rumen, unsaturated fatty acids are toxic to rumen microorganisms, and in self-defense, microorganisms perform biohydrogenation that converts unsaturated fatty acids into saturated fatty acids. Forages have a high content of polyunsaturated fatty acids in their composition [64]. In vitro studies concluded that the increase in dietary C18:2 polyunsaturated fatty acid inhibits the biohydrogenation of C18:2 polyunsaturated fatty acid to C18:0 saturated fatty acid [65,66]. This could be an explanation for the higher value of polyunsaturated fatty acids in the meat of the control animals that contained 80% of Tifton-85 in the animals’ diet (Table 5), providing greater intestinal absorption of polyunsaturated fatty acids, increasing the content of fatty acids of the n3 family (Table 5), and thus increasing the concentration of omega-3 in the meat of animals fed this diet (*p* < 0.05).

A high omega-6:omega-3 ratio is associated with risk of atherosclerosis or coronary heart disease [67]. For this variable, values below 4.0 are desirable for disease prevention [68]. In the present study, this ratio was 4.11 in meat from animals fed the control diet, 2.9 and 3.0 in meat from animals fed a diet with 25 and 55% forage cactus, respectively (Table 5). Therefore, goats that receive forage cactus in their diet have a reduction in the omega-6:omega-3 ratio in their meat, which is beneficial for human health.

### 4.4. Sensory Analysis of Meat

The influence of diets rich in polyunsaturated fatty acids on the volatile compounds of beef and sheep show greater lipid oxidation in meat [69]. In the present study, the meat of the animals that received the control diet had a higher content of polyunsaturated fatty acids (Table 5), which may have provided greater lipid oxidation in the meat, resulting in greater rancidity and lower acceptance by consumers (Table 6). Lipids, especially unsaturated fatty acids, are prone to oxidation and can act as pro-oxidants, resulting in the development of off-flavors [70]. The sensory attributes that presented the highest scores, by consumers, were the meat of goats that received cactus in the diet (Table 6), and it is important to consider the evaluation of quality and consumer preference in animal production.

## 5. Conclusions

Goats fed cactus pear had better meat texture compared to animals fed Tifton 85 hay in their diet. There is no influence of the other analyzed parameters of physical quality of the meat in relation to the type of diet supplied to the goats in this research. Meat from goats fed with the inclusion of 55% of cactus pear in the diet showed more favorable sensory attributes by consumers than meat from animals fed other diets. Therefore, it is recommended that Tifton 85 hay is replaced with 55% forage cactus, as it provides lower lipid content and higher content of monounsaturated fatty acids in goat meat, resulting in an excellent option for consumers who mainly seek healthier food.

## Figures and Tables

**Table 1:** Chemical composition of diets ingredients in a dry matter basis.

Ingredients (g/kg DM)	Forage Cactus	Tifton 85 Hay	Ground Corn Grain	Soybean Meal
Dry matter	117.30	886.90	870.10	874.80
Mineral matter	92.30	65.00	11.70	70.10
Organic matter	907.70	935.00	988.30	929.90
Crude protein	38.50	98.30	96.70	513.20
Ether extract	34.80	19.40	45.30	23.10
Neutral detergent fiber ^1^	186.70	691.10	90.60	131.80
Acid detergent fiber	102.00	362.80	50.00	84.90
Total carbohydrates	834.30	817.40	846.30	393.60
Non-fibrous carbohydrates	647.60	126.30	755.70	261.80

^1^ Neutral detergent fiber corrected for ash and protein. DM = dry matter.

**Table 2:** Ingredients and chemical composition and fatty acid profile of experimental diets in a dry matter basis.

Ingredients (g/kg DM)	Diets ^1^		
	Control	25%	55%
Tifton 85 hay	800.00	550.00	250.00
Ground corn grain	50.00	35.00	45.00
Soybean meal	130.00	145.00	130.00
Forage cactus	0.00	250.00	550.00
Urea	10.00	10.00	15.00
Mineral salt	10.00	10.00	10.00
Bromatological composition			
Dry matter	884.50	335.80	192.40
Mineral matter	71.70	79.40	86.70
Organic matter	918.30	910.60	898.30
Crude protein	178.30	169.60	159.00
Ether extract	20.80	24.30	29.00
Neutral detergent fiber ^2^	574.50	449.10	296.70
Acid detergent fiber	303.80	239.10	160.10
Total carbohydrates	747.40	744.80	752.50
Non-fibrous carbohydrates	172.80	295.80	455.80
Fatty acid profile (%)			
C14:0	1.33	2.56	4.09
C16:0	16.65	18.71	21.07
C16:1	1.28	2.27	3.43
C18:0	3.23	5.12	7.31
C18:1 cis-9	8.97	9.99	11.35
C18:1 trans-9	4.35	4.55	4.78
C18:2 cis-6	6.97	6.00	5.00
C18:2 trans-6	30.57	27.88	24.05
C18:3 α-linolenic	6.60	4.93	2.97
C20:0	2.40	1.74	0.94
C24:0	2.00	1.58	1.06

^1^ Control = 80% Tifton 85 hay and 20% concentrate, 25% = 55% Tifton 85 hay, 25% forage cactus and 20% concentrate, 55% = 25% Tifton 85 hay, 55% forage cactus and 20% concentrate. ^2^ Neutral detergent fiber corrected for ash and protein. DM = dry matter.

**Table 3:** Physical quality of the *Longissimus lumborum* muscle of goats fed forage cactus as a substitute for Tifton 85 hay.

Physical Parameters	Diets ^1^			SEM ^2^	*p* Value
	Control	25%	55%		
pH	5.97	5.87	5.95	0.06	0.26
Cooking losses (%)	29.71	32.31	29.31	2.19	0.35
Water holding capacity (%)	80.67	77.91	77.17	0.86	0.24
Shear force (N) ^3^	85.41 a	63.44 b	65.80 b	0.73	0.08
Luminosity (L*)	42.14	42.27	40.63	1.59	0.53
Redness (a*)	19.72	19.83	20.05	0.88	0.93
Yellowness (b*)	7.88	8.12	7.56	0.90	0.82
Tilt angle (h*)	21.56	22.22	20.51	2.20	0.74
Croma (C*)	21.30	21.47	21.52	0.98	0.97

^1^ Control = 80% Tifton 85 hay and 20% concentrate, 25% = 55% Tifton 85 hay, 25% forage cactus and 20% concentrate, 55% = 25% Tifton 85 hay, 55% forage cactus and 20% concentrate. ^2^ SEM = Standard error of the mean. ^3^ N = Warner–Bratzler shear force (Newton force). Values followed by distinct letters on the same line differ significantly from each other.

**Table 4:** Chemical composition of the *Longissimus lumborum* muscle of goats fed forage cactus as a substitute for Tifton 85 hay.

Chemical Composition (%)	Diets ^1^			SEM ^2^	*p* Value
	Control	25%	55%		
Moisture	77.57 a	76.33 ab	76.11 b	0.41	0.0052
Proteins	20.43	20.68	20.33	0.43	0.71
Lipids	1.56 a	1.33 b	1.26 c	0.04	0.001
Mineral matter	1.16	1.14	1.17	0.02	0.57

^1^ Control = 80% Tifton 85 hay and 20% concentrate, 25% = 55% Tifton 85 hay, 25% forage cactus and 20% concentrate, 55% = 25% Tifton 85 hay, 55% forage cactus and 20% concentrate. ^2^ SEM = Standard error of the mean. Values followed by distinct letters on the same line differ significantly from each other by the Tukey test at 5% probability.

**Table 5:** Fatty acid profile of the *Longissimus lumborum* muscle of goats fed forage cactus as a substitute for Tifton 85 hay.

Fatty Acids (%)	Diets ^1^			SEM ^2^	*p* Value
	Control	25%	55%		
Saturated					
C4:0	0.01	0.01	0.1	0.03	0.15
C6:0	0.09	0.02	0.08	0.04	0.16
C8:0	0.02 a	0.01 ab	0.01 b	0.003	0.02
C10:0	0.09 b	0.12 a	0.12 a	0.01	0.05
C11:0	0.02	0.01	0.01	0.006	0.61
C12:0	0.04	0.05	0.05	0.005	0.68
C13:0	0.02 a	0.01 b	0.01 b	0.003	0.07
C14:0	0.77 b	1.32 ab	1.38 a	0.11	0.001
C15:0	0.32 a	0.28 b	0.28 b	0.01	0.08
C16:0	21.01 b	23.34 a	22.95 a	0.64	0.05
C17:0	1.26	1.17	1.20	0.06	0.59
C18:0	13.69	12.53	12.26	0.61	0.26
C20:0	0.06	0.05	0.04	0.004	0.61
C21:0	0.003	0.005	0.003	0.002	0.74
C22:0	0.004	0.005	0.009	0.004	0.64
C23:0	0.006	0.004	0.004	0.002	0.88
C24:0	2.47 a	1.34 ab	0.71 b	0.32	0.005
Total saturates	40.21	40.36	39.68	0.85	0.84
Monounsaturated					
C14:1	0.02 b	0.05 ab	0.06 a	0.08	0.003
C15:1 *cis*-10	0.01	0.002	0.008	0.0004	0.26
C16:1	1.21 b	1.58 ab	1.76 a	0.09	0.002
C17: 1 *cis*-10	0.89	1.01	1.10	0.07	0.18
C18:1*cis* n9	33.16 b	43.35 ab	46.86 a	1.70	0.002
C18:1*trans* n9	2.29	2.18	2.65	0.25	0.41
C20:1n9 *cis*-11	0.13	0.09	0.09	0.01	0.31
C22:1n9	0.002	0.004	0.004	0.003	0.66
C24:1	0.009	0.002	0.01	0.004	0.13
Total monounsaturated	37.75 b	48.32 ab	52.17 a	1.79	0.001
Polyunsaturated					
C18:2 *cis* n-6	6.38 a	4.26 ab	2.94 b	0.38	0.001
C18:2 *trans* n-6	0.06 b	0.07 b	0.40 a	0.13	0.06
C18:3n-3	0.73 a	0.54 ab	0.26 b	0.04	0.001
C18:3n-6	0.08 a	0.05 ab	0.04 b	0.01	0.05
C20:2	0.80a	0.43b	0.47b	0.10	0.08
C20:3n-3	0.004	0.01	0.005	0.003	0.55
C20:3n-6	0.65 a	0.32 ab	0.21 b	0.08	0.01
C20:4n-6	9.71 a	3.77 ab	2.49 b	1.18	0.003
C20:5n-3	2.18 a	1.24 ab	0.47 b	0.78	0.001
C22:2	0.03	0.09	0.06	0.02	0.32
C22:6n-3	0.59 a	0.30 ab	0.03 b	0.13	0.001
Total polyunsaturated	21.44 a	11.16 ab	6.90 b	2.17	0.001
Total unsaturated	59.72	59.55	60.33	0.88	0.81
Omega-3	4.16 a	2.93 ab	1.83 b	0.23	0.001
Omega-6	17.47	8.52	6.07	6.57	0.15
Omega- 6:omega-3	4.11 a	2.90 b	3.00 b	0.35	0.08
Monounsaturated:saturated	0.93 b	1.19 ab	1.32 a	0.05	0.006
Polyunsaturated:saturated	0.53 a	0.28 ab	0.17 b	0.05	0.001

^1^ Control = 80% Tifton 85 hay and 20% concentrate, 25% = 55% Tifton 85 hay, 25% forage cactus and 20% concentrate, 55% = 25% Tifton 85 hay, 55% forage cactus and 20% concentrate. ^2^ SEM = Standard error of the mean. Values followed by distinct letters on the same line differ significantly from each other by the Tukey test at 5% probability.

**Table 6:** Sensory analysis of the *Longissimus lumborum* muscle of goats fed forage cactus as a substitute for Tifton 85 hay.

Sensory Attributes	Diets ^1^			SEM ^2^	*p* Value
	Control	25%	55%		
General appearance	6.40 b	7.07 a	7.13 a	0.237	0.0034
Aroma	5.84 b	6.48 a	6.62 a	0.281	0.0135
Flavor	5.99 b	6.68 a	6.81 a	0.300	0.0138
Texture	5.79b	6.30 b	7.07 a	0.301	0.0001
Juiciness	5.69 b	6.35 a	6.81 a	0.303	0.0012

^1^ Control = 80% Tifton 85 hay and 20% concentrate, 25% = 55% Tifton 85 hay, 25% forage cactus and 20% concentrate, 55% = 25% Tifton 85 hay, 55% forage cactus and 20% concentrate. ^2^ SEM = Standard error of the mean. Values followed by distinct letters on the same line differ significantly from each other by the Tukey test at 5% probability.

## Data Availability

The dada presented in this study are available on request from the corresponding author.
